# Robust circadian rhythms in organoid cultures from PERIOD2::LUCIFERASE mouse small intestine

**DOI:** 10.1242/dmm.014399

**Published:** 2014-07-04

**Authors:** Sean R. Moore, Jill Pruszka, Jefferson Vallance, Eitaro Aihara, Toru Matsuura, Marshall H. Montrose, Noah F. Shroyer, Christian I. Hong

**Affiliations:** 1 Gastroenterology, Hepatology and Nutrition, Cincinnati Children’s Hospital Medical Center, University of Cincinnati, OH 45229-3039, USA; 2 Molecular and Cellular Physiology, University of Cincinnati College of Medicine, OH 45267-0576, USA

**Keywords:** Circadian rhythm, Intestinal organoid, PERIOD2, R-spondin, RU486

## Abstract

Disruption of circadian rhythms is a risk factor for several human gastrointestinal (GI) diseases, ranging from diarrhea to ulcers to cancer. Four-dimensional tissue culture models that faithfully mimic the circadian clock of the GI epithelium would provide an invaluable tool to understand circadian regulation of GI health and disease. We hypothesized that rhythmicity of a key circadian component, PERIOD2 (PER2), would diminish along a continuum from *ex vivo* intestinal organoids (epithelial ‘miniguts’), nontransformed mouse small intestinal epithelial (MSIE) cells and transformed human colorectal adenocarcinoma (Caco-2) cells. Here, we show that bioluminescent jejunal explants from PERIOD2::LUCIFERASE (PER2::LUC) mice displayed robust circadian rhythms for >72 hours post-excision. Circadian rhythms in primary or passaged PER2::LUC jejunal organoids were similarly robust; they also synchronized upon serum shock and persisted beyond 2 weeks in culture. Remarkably, unshocked organoids autonomously synchronized rhythms within 12 hours of recording. The onset of this autonomous synchronization was slowed by >2 hours in the presence of the glucocorticoid receptor antagonist RU486 (20 μM). Doubling standard concentrations of the organoid growth factors EGF, Noggin and R-spondin enhanced PER2 oscillations, whereas subtraction of these factors individually at 24 hours following serum shock produced no detectable effects on PER2 oscillations. Growth factor pulses induced modest phase delays in unshocked, but not serum-shocked, organoids. Circadian oscillations of PER2::LUC bioluminescence aligned with *Per2* mRNA expression upon analysis using quantitative PCR. Concordant findings of robust circadian rhythms in bioluminescent jejunal explants and organoids provide further evidence for a peripheral clock that is intrinsic to the intestinal epithelium. The rhythmic and organotypic features of organoids should offer unprecedented advantages as a resource for elucidating the role of circadian rhythms in GI stem cell dynamics, epithelial homeostasis and disease.

## INTRODUCTION

Circadian rhythms are biological processes with ~24-hour oscillations that regulate diverse life functions across a wide spectrum of organisms (Panda et al., 2002). These processes include the digestive, immune and regenerative functions of the gastrointestinal (GI) epithelium, which are necessary for organismal growth and survival (Potten et al., 1977; Hoogerwerf, 2006; Hussain and Pan, 2009; Karpowicz et al., 2013; Yu et al., 2013). In humans, disruption of circadian rhythms is increasingly common and associated with a variety of GI diseases ranging from diarrhea to ulcers to cancer (Savarino et al., 1996; Hoogerwerf, 2009; Xu et al., 2013). Progress in understanding the mechanisms by which circadian rhythms influence GI health and disease has lagged, in part, due to a paucity of *in vitro* models with appropriate phenotypes and robust circadian rhythms (Hughes et al., 2009). For example, Caco-2 cells are a commonly studied human colorectal adenocarcinoma cell line that has recently been shown to exhibit circadian oscillations of circadian component PERIOD2 (PER2) (Ballesta et al., 2011; Swanson et al., 2011); however, the limited capacity of Caco-2 cells for differentiation and their dampened circadian oscillations over time might hinder extrapolation of *in vitro* results to functional GI tissue (Hughes et al., 2009). To our knowledge, a tissue culture system that overcomes these limitations has yet to be described.

Intestinal organoids have recently emerged as a powerful platform for understanding adult stem cell dynamics, intestinal epithelial homeostasis and gut pathophysiology (Sato et al., 2009; reviewed by Sato and Clevers, 2013). Mouse intestinal organoids arise from *Lgr5*+ intestinal stem cells that differentiate into all lineages present in the epithelium and, strikingly, self-organize into three-dimensional structures with crypt-, villous- and lumen-like domains (Sato et al., 2009). We hypothesized that rhythmicity of PER2 would diminish in amplitude along a continuum from mouse small intestinal explants, intestinal organoids, a nontransformed mouse small intestinal epithelial (MSIE) cell line (Whitehead et al., 1993) and transformed human colorectal adenocarcinoma cells (Caco-2). Here we show that: (1) jejunal explants and organoids derived from PERIOD2::LUCIFERASE fusion protein (PER2::LUC) mice (Yoo et al., 2004) display robust circadian rhythms, (2) PER2::LUC organoids synchronize their circadian clock in response to serum shock, (3) unshocked organoids autonomously synchronize their circadian clock within 12 hours of monitoring and (4) organoid growth factors and Matrigel™ enhance circadian rhythms.

## RESULTS

**Fig. 1.**
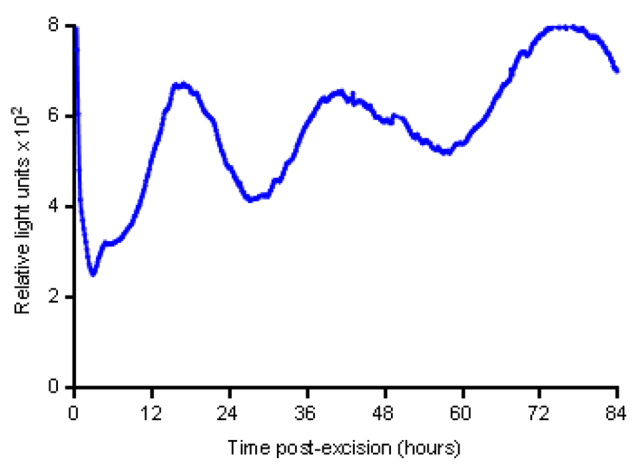
**Circadian rhythms in PER2::LUC jejunal explants.** Representative jejunal segments displaying 1.5- to 2-fold circadian PER2 oscillations for up to 3 days following excision, with lengthening of the period of PER2 oscillations over time.

**Fig. 2.**
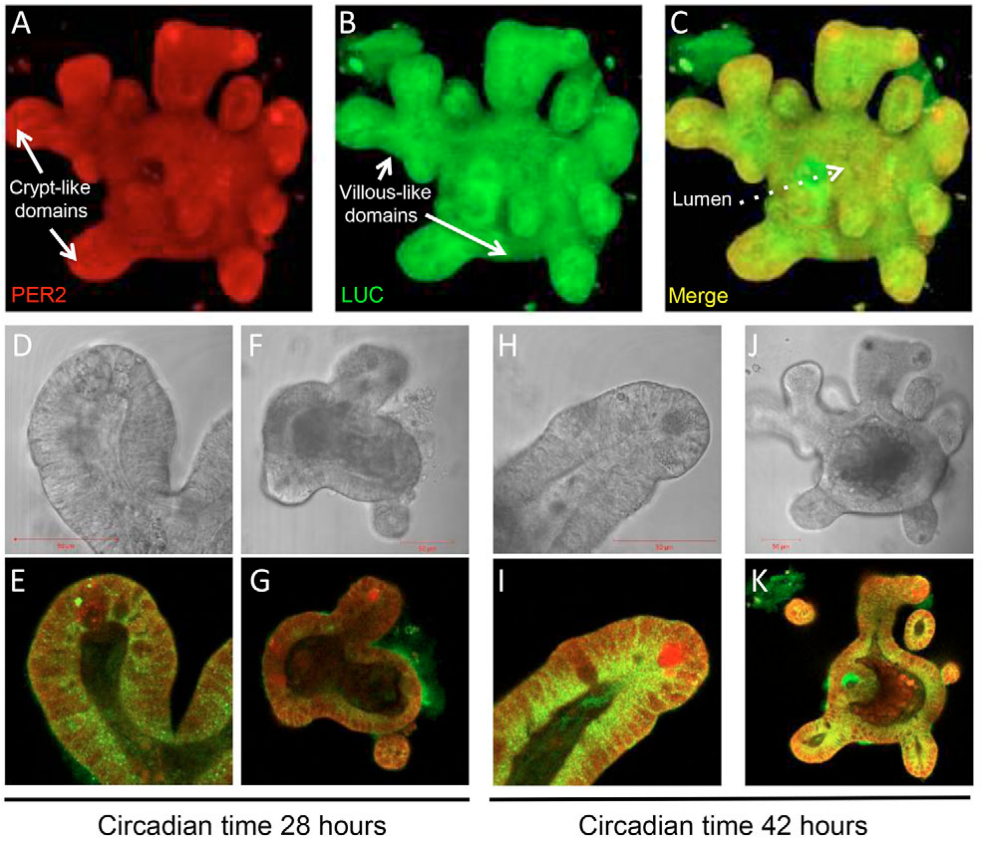
**Organotypic PER2::LUC jejunal organoids display circadian variation in PER2 and LUC expression.** (A–C) Representative three-dimensional confocal image reconstruction of a PER2::LUC organoid with crypt, villous and lumen domains 42 hours after serum shock and staining with fluorescent antibodies against PER2 and LUC throughout. Comparison of single plane confocal images at (D–G) 28 hours following serum shock versus (H–K) 42 hours following serum shock reveals a time-dependent increase in PER2 and LUC staining.

**Fig. 3.**
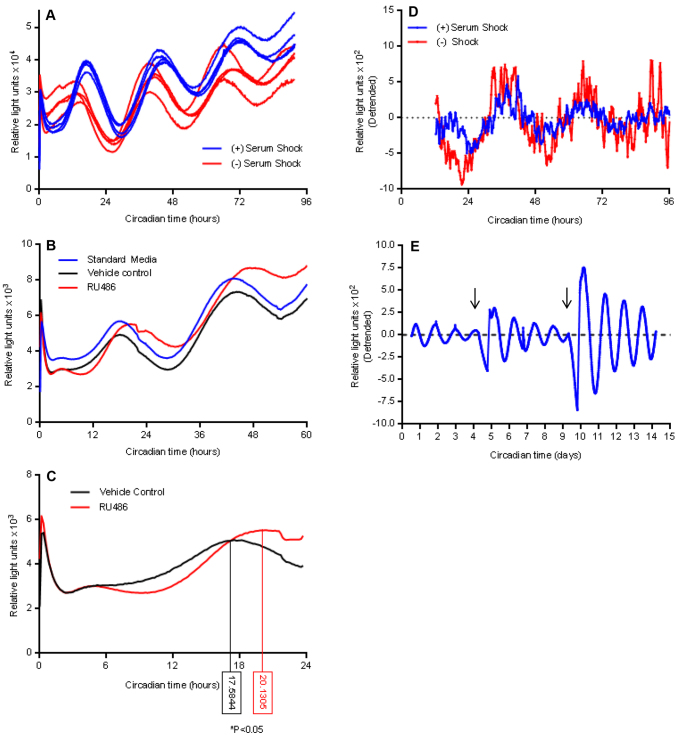
**Robust circadian rhythms in PERIOD2::LUCIFERASE jejunal organoids.** (A) PER2::LUC organoids display 1.5- to 3-fold circadian oscillations of PER2 abundance following serum shock (blue), as measured by luciferase bioluminescence. In the absence of serum shock (red), PER2::LUC organoids develop synchronized PER2 oscillations within 8 to 12 hours of recording (*n*=4 per group). (B) Representative traces of detrended luciferase bioluminescence of unshocked organoids that had been treated with the steroid antagonist RU486 (20 μM, red) or an ethanol vehicle control (black). Standard medium is shown in blue. (C) Averaged tracings of detrended luciferase bioluminescence in unshocked PER2::LUC jejunal enteroids that had been treated with either the steroid receptor antagonist RU486 (red) or an ethanol vehicle control (blue). Treatment with RU486 delays the initial peak of PER2 oscillations by a mean of 2.1 hours (*P<0.0001 by Mann–Whitney *U* test; *n*=9 samples per group). Numbers in boxes indicate the circadian time at which the peak PER2 oscillation is reached. (D) Representative tracings of detrended luciferase bioluminescence in shocked (blue) and unshocked (red) organoids in the absence of Matrigel™. PER2 oscillations appear more variable and lower in amplitude but maintain a clear circadian rhythm. (E) Replenishment of organoid medium and luciferin every 5 days enhances and extends PER2 oscillations beyond 2 weeks but resets oscillations after day 10 (representative detrended tracings).

In initial experiments, jejunal explants from PER2::LUC mice displayed 1.5- to 2-fold circadian oscillations of PER2 protein abundance that persisted for up to ~84 hours (Fig. 1), with a notable lengthening of period over time. We next generated organoids derived from the jejunal crypts of PER2::LUC mice. A representative three-dimensional confocal image reconstruction of a jejunal PER2::LUC organoid at 42 hours following serum shock, with PER2 and LUC fluorescent antibody staining throughout all crypt- and villous-like domains is shown in Fig. 2A–C. The single plane confocal images in Fig. 2D–K show increased PER2 and LUC staining at 42 hours versus 28 hours following serum shock, indicating a circadian variation in the expression of these proteins. Separately, the incubating luminometer real-time measurements of PER2 abundance in PER2::LUC jejunal organoids demonstrated 1.5- to 3-fold circadian oscillations of PER2 abundance for up to 96 hours following serum shock (Fig. 3A). In addition to a longer duration of detectable PER2 rhythms, period lengthening over time appeared less pronounced in organoids versus explants.

**RESOURCE IMPACT**

**Background**

Circadian rhythms regulate physiological functions of the gastrointestinal (GI) epithelium but also influence the risk and outcome of several GI diseases, ranging from diarrhea to ulcers to cancer. Circadian rhythms also mediate the absorption and GI side effects of numerous drugs. Reliable intestinal *in vitro* models with appropriate phenotypes and robust circadian rhythms are currently limited. Four-dimensional tissue culture models with organotypic features and the ability to reproduce the circadian rhythms of the GI epithelium would help advance our understanding of how circadian rhythms regulate GI health and disease.

**Results**

In this study, the authors used transgenic mice expressing bioluminescent luciferase-labeled PERIOD2 (PER2), a key circadian clock component. PER2::LUCIFERASE (PER2::LUC) intestinal segments showed robust circadian rhythms of PER2 protein abundance for up to 3 days following excision from mice. Subsequently, the authors generated intestinal organoids (epithelial ‘mini-guts’) by culturing jejunal crypts derived from PER2::LUC mice. Bioluminescent PER2::LUC organoids showed structural features typical of the intestinal epithelium and exquisite circadian rhythms; these rhythms synchronized in response to a high concentration of serum (serum shock), a typical external cue designed to align the clocks of mammalian cells in culture. Interestingly, even in the absence of serum shock, PER2::LUC organoids demonstrated the unusual ability to self-synchronize circadian rhythms after 12 hours in culture. Moreover, in this tissue culture model, the circadian patterns of *Per2* gene expression over 48 hours were more robust as compared with serum-shocked intestinal epithelial cell lines from mice (mouse small intestine epithelial cells) and humans (Caco-2 colorectal adenocarcinoma cells).

**Implications and future directions**

Because of their robust circadian rhythms and other organotypic features, bioluminescent organoids offer an exciting new tool to understand the interaction of circadian rhythms with stem cell dynamics and epithelial homeostasis in the GI tract. Such interplay might have broad implications for disease modeling, stem-cell-based therapies, personalized medicine and drug development. Organoids with intrinsic circadian rhythms might provide a more sensitive *in vitro* assay to test the absorption, mechanisms and GI toxicity of new drugs. In addition, the development of intestinal organoids derived from human tissue should advance the translation of *in vitro* findings into therapeutic improvements for human GI diseases.

Mammalian cell lines derived from peripheral tissues, such as the gut, require an extrinsic cue, such as serum shock or steroid pulse, to synchronize circadian rhythms (Balsalobre et al., 1998). We compared PER2 oscillations in serum-shocked and unshocked organoids. We found that serum shocked organoids displayed synchronized PER2 oscillations immediately upon initiation of bioluminescent recordings (Fig. 3A). Strikingly, unshocked organoids displayed modest PER2 rhythms during the first 8 to 12 hours of recording then spontaneously developed robust PER2 oscillations out of phase with shocked controls (Fig. 3A). The steroid receptor antagonist RU486 has been shown to disrupt circadian rhythms in mesenchymal stem cells (So et al., 2009); therefore, we tested the effects of RU486 on organoid PER2 rhythms in the absence of serum shock. Pre-treatment of organoids with RU486 (20 μM) significantly delayed the time to the first peak of PER2 abundance by 2 hours (*P*<0.0001; Fig. 3B,C). Separately, PER2::LUC organoids suspended in medium without Matrigel™ showed dramatic reductions in the amplitude and persistence of PER2 oscillations, but maintained circadian rhythms both in the presence and absence of serum shock (Fig. 3D). Furthermore, by refreshing organoid medium and adding luciferin every 5 days (arrows, Fig. 3E), we were able to extend the duration of detectable circadian PER2 oscillations up to 15 days, with a reset of PER2 oscillations following the second medium change at day 10.

**Fig. 4.**
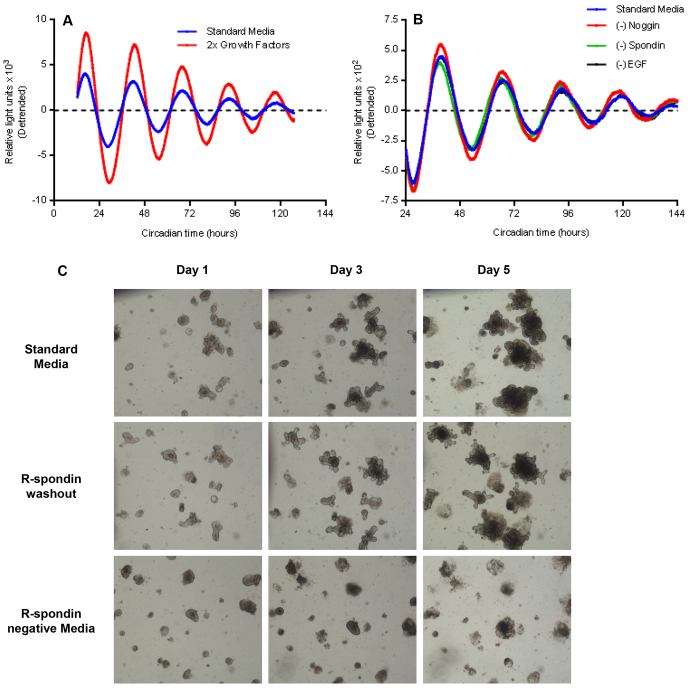
**Effects of organoid growth factors on PER2 rhythms.** (A) Doubling standard concentrations of EGF, Noggin and R-spondin in organoid medium increases the amplitude of PER2 oscillations (representative detrended tracings). (B) Subtraction of these growth factors individually from organoid medium 24 hours following serum shock does not dampen PER2 rhythms compared with standard medium (blue). Representative detrended tracings are shown. (C) Top panels, sequential micrographs (4× magnification) of serum-shocked organoids passaged into standard medium over 5 days. Middle panels, organoids with R-spondin washout at 24 hours following serum shock. Bottom three panels, organoids passaged immediately into R-spondin-negative medium following serum shock show loss of proliferation and death by day 5.

To optimize conditions favorable for circadian rhythms in organoids, we doubled standard concentrations of EGF, Noggin and R-spondin in organoid medium and found a resulting increase in the amplitude of PER2 oscillations following serum shock (Fig. 4A). To test the effects of depletion of individual factors on organoid circadian rhythms, while maintaining organoid viability, we subtracted these three growth factors individually from organoid medium at 24 hours following serum shock. We detected no significant differences in either the amplitude or duration of PER2 oscillations (Fig. 4B).

Under typical culture conditions, the removal of R-spondin from organoid growth medium induces growth arrest and cell death within 2 to 4 days. We therefore compared the growth of passaged organoids over 5 days under the following conditions: (1) standard Sato medium following serum shock, (2) Sato medium with removal of R-spondin at 24 hours following serum shock (i.e. the R-spondin-negative experimental condition in Fig. 4B), and (3) Sato medium with removal of R-spondin immediately following serum shock. Organoids that had been maintained in R-spondin-free medium immediately following serum shock showed marked reductions in both size and number relative to controls by day 3, indicating growth arrest and death. By contrast, organoids maintained in Sato medium with removal of R-spondin at 24 hours following serum shock remained similar in size and numbers to controls at day 5, but exhibited an atypical lengthening of crypt-like domains. When organoids from all three conditions were passaged into standard minigut medium at the end of day 5, only control organoids that had been continuously maintained in R-spondin (top panels, Fig. 4C) before passaging formed new organoids. Taken together, these results suggest that the R-spondin-negative organoids used in Fig. 4B were sufficiently viable to allow continued measurements of circadian rhythms, but lacked the regenerative capacity to form new organoids upon passaging.

**Fig. 5.**
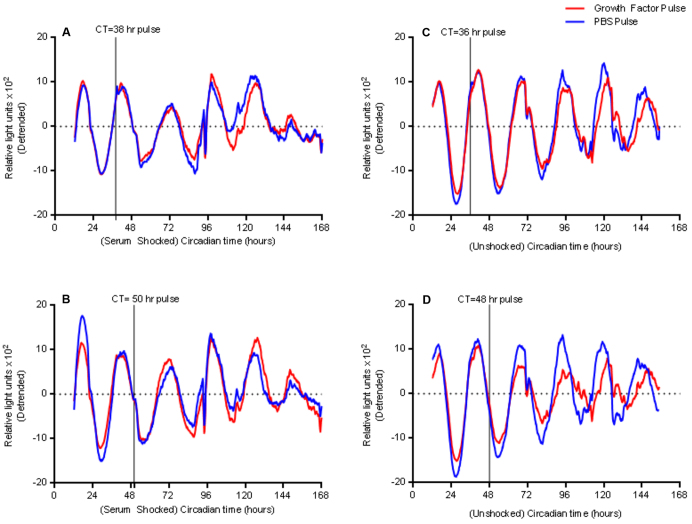
**Growth factors induce phase shifts in PER2 oscillations in unshocked organoids.** (A,B) Serum-shocked organoids show no response to growth factors (red) pulsed at either an (A) upslope or (B) downslope of PER2 oscillations versus a PBS vehicle control (blue). By contrast, unshocked enteroids that were pulsed with growth factors (red) at an (C) upslope or (D) downslope of PER2 oscillations both show phase shifts by day 5 of recording (representative detrended tracings). CT, circadian time.

As noted above, a medium change at day 10 of bioluminescent recordings reset PER2 oscillations (Fig. 3E), therefore we tested whether a combined pulse of the growth factors EGF, Noggin and R-spondin altered PER2 oscillations at different phases of the circadian clock. In serum-shocked organoids (Fig. 5A,B), growth factor pulses at circadian times 38 hours and 50 hours did not perturb PER2 oscillations. By contrast, unshocked organoids (Fig. 5C,D) that had been pulsed with growth factors at circadian times 36 and 48 hours showed modest phase delays in PER2 oscillations relative to controls, more so following the pulse at circadian time 48 hours.

**Fig. 6.**
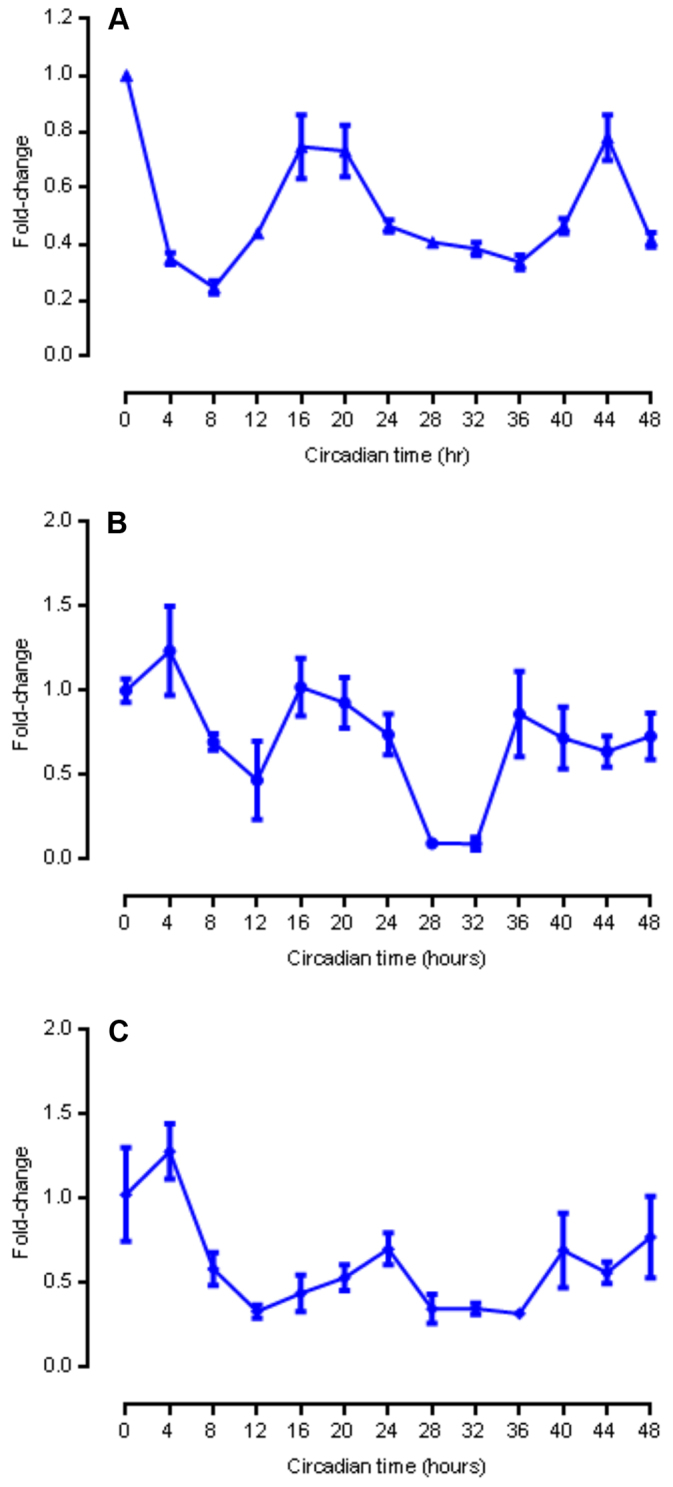
**Circadian oscillations of *Per2* mRNA expression in PER2::LUC organoids and cell lines.** (A) *Per2* mRNA expression over 48 hours in mouse small intestinal organoids (*n*=3 per time point). (B) *Per2*mRNA expression over 48 hours in nontransformed mouse small intestinal epithelial (MSIE) cells under quiescent conditions (*n*=3 per time point). (C) *Per2* mRNA expression over 48 hours in transformed human colorectal adenocarcinoma Caco-2 cells (*n*=3 per time point). Means±s.e.m. are shown.

To compare circadian rhythms of *Per2* gene expression in PER2::LUC organoids, MSIE cells and Caco-2 cells, we performed quantitative (q)PCR over 48 hours at 4-hour time resolution under standard culture conditions. Serum-shocked PER2::LUC organoids exhibited synchronized circadian oscillations of *Per2* gene expression (Fig. 6A) over 48 hours. Serum-shocked MSIE and Caco-2 cells exhibited less consistent circadian oscillations of *Per2* over 48 hours (Fig. 6B,C, respectively) relative to organoids.

## DISCUSSION

To our knowledge, this study is the first to demonstrate robust oscillations of a core circadian clock component in small intestine explants and organoid preparations, providing additional evidence for a self-regulating, peripheral clock that is intrinsic to the intestinal epithelium. By identifying a novel organotypic feature of intestinal organoids, this study also provides a new resource to elucidate the role of circadian rhythms in adult stem cell dynamics, intestinal epithelial homeostasis and gut pathophysiology. As such, bioluminescent organoid platforms offer several potential advantages over two-dimensional intestinal cell culture. First, organoids are a nontransformed cell culture system and retain more *in vivo* characteristics, including the capacity to differentiate and organize into crypt- and villous-like domains. In addition, the simultaneous presence of intestinal stem cells and their progeny within the organized three-dimensional structure of the organoids is likely to provide a more accurate model of the cell-cell contacts and communication pathways that coordinate circadian behaviors of the intestinal epithelium *in vivo*. Finally, luciferase bioluminescent organoids allow real-time measurement of PER2 abundance at high sampling frequencies (every 10 minutes). Although highly sensitive and specific, qPCR analyses of homogenates from repeated harvests of PER2::LUC organoids, MSIE and Caco-2 cells was comparably less efficient for experiments requiring fine time-resolution measurements over several days.

We observed a lengthening of PER2 periods over 72 hours of bioluminescent recording in jejunal explants. Period lengthening has previously been demonstrated in PER2::LUC colon explants and is probably due to compromised tissue viability *ex vivo* (Malloy et al., 2012). By contrast, jejunal organoids appeared to maintain a ~24-hour period for up to 96 hours of bioluminescent recording, with no change in medium.

Our finding that PER2::LUC organoids autonomously synchronize circadian rhythms is surprising, but correlates well with our finding of PER2 oscillations in unshocked jejunal explants. This distinguishes the circadian behavior of organoids from transformed mammalian cell lines, such as NIH 3T3 cells, which require a serum shock in order to synchronize (Nagoshi et al., 2004). The organoid cultures we studied contained multiple organoids, suggesting that not only do individual organoids self-synchronize but that populations of organoids within culture also achieve synchrony. Matrigel™, although not required for organoid synchronization, clearly strengthened the amplitude of PER2 rhythms. It is possible that Matrigel™ contains synchronizing agents that are yet to be identified or that it promotes organoid stability and cell-cell communication. Present within organoids, enteroendocrine cells represent a potential source of secreted factors that might entrain organoid rhythms. Enteroendocrine cells produce a variety of gut hormones, including vasoactive intestinal peptide, which has been shown to synchronize the circadian clock in neurons of the suprachiasmatic nucleus (Aton et al., 2005). The extent to which enteroendocrine cells or other epithelial cell lineages entrain gut epithelial clocks in organoids and *in vivo* is unknown and will be an important question for future research.

Interestingly, a doubling of the concentration of growth factors required for organoid culture (EGF, Noggin and R-spondin) enhanced the amplitude of PER2 rhythms (perhaps by increasing organoid size and number); however, these growth factors were not individually required for maintenance of PER2 rhythms once oscillations were established. We speculate that endogenous production of growth factors following their removal from medium 24 hours following serum shock might be sufficient to maintain short-term viability and circadian rhythms in established organoid cultures. Our finding that growth factor pulses delay the phase of autonomously synchronized organoids is intriguing. *In vivo*, Paneth cells provide these growth factors, thereby constituting the intestinal stem niche (Sato et al., 2011). Well-described circadian rhythms of mouse crypt base columnar stem cell proliferation (Potten et al., 1977) suggest that the cellular dynamics of such growth factors is almost certainly under circadian regulation and should thus be further explored.

The close correlation of PER2 oscillations in jejunal explants and organoids emphasizes potential advantages of intestinal organoids as a relevant experimental platform for understanding temporal regulation of intestinal epithelial homeostasis and stem cell dynamics and differentiation in mammals. In *Drosophila*, Karpowicz and colleagues have recently reported that intestinal stem cell regeneration is rhythmic and regulated by the circadian clock and that circadian clock mutations impair the regenerative capacity of intestinal stem cells (Karpowicz et al., 2013). Work is underway to confirm these findings in mice and we propose that intestinal organoids will provide an ideal four-dimensional tissue culture system in which to study these phenomena. The circadian properties of organoids will also be relevant to exploring recently uncovered connections between gut microbiota, circadian rhythms and epithelial homeostasis in isolated intestinal epithelial cells (Mukherji et al., 2013). Finally, recent breakthroughs with the development of intestinal organoids derived from human stem cells and gut tissue should accelerate translation of these findings into improvements in human health (Spence et al., 2011).

## MATERIALS AND METHODS

### Intestinal explants and organoids

We obtained PER2::LUC mice on a C57Bl/6J background from Jackson Laboratories (Bar Harbor, ME, USA; Yoo et al., 2004). PER2::LUC mice and wild type C57Bl/6j mice were housed in a barrier facility with an ambient temperature of 22°C, a relative humidity ranging from 30 to 70% and a 14-hour light:10-hour dark cycle. For *ex vivo* explant experiments, PER2::LUC mice were killed 3 hours after lights on using CO_2_ inhalation followed by cervical dislocation according to rules and regulations of the Institutional Animal Care and Use Committee at Cincinnati Children’s Hospital Medical Center. The jejunum was harvested and flushed with cold Kreb’s solution before being cut into 3–5-mm segments. Tissue was splayed open and placed lumen side up onto a Millicell™ cell culture insert (EMD Millipore Corporation, Billerica, MA, USA) in a 35-mm dish. Within 30 minutes of killing mice, explants were placed in a Kronos Dio™ AB-2550 incubating luminometer (ATTO Corporation, Tokyo, Japan) with Dulbecco’s modified Eagle’s medium and 200 μM Beetle Luciferin K^+^ salt (Promega, Fitchburg, WI, USA) for real-time periodic quantification of PER2 protein abundance by bioluminescence recording (Malloy et al., 2012).

Using the methods of Sato and colleagues (Sato et al., 2009), we prepared intestinal organoids (enteroids) by isolating fresh mid-jejunal crypts from PER2::LUC mice. We killed animals using CO_2_ inhalation followed by cervical dislocation. Approximately 6 cm of jejunum was dissected from the mouse, flushed with ice cold PBS and splayed open with scissors. The jejunal segment was then sliced into 1-cm pieces and transferred to 5 ml of cold PBS on ice. This suspension was placed on a rocking table at 4°C for 5 minutes to remove residual blood or stool from jejunal segments. After rocking, the PBS was aspirated and 5 ml of 2 mM EDTA chelation buffer was added. The suspension was placed back on a rocking table at 4°C for 30 minutes. Chelation buffer was then removed and 5 ml of shaking buffer (PBS with 43.3 mM sucrose and 54.9 mM sorbitol) was added. The conical tube was gently shaken by hand for 2 minutes. A sample of the crypt suspension was visualized by using a microscope to ensure crypts had been released. If crypts were still attached, samples were gently shaken for an additional 30 seconds to 2 minutes. The intestinal crypt suspension was filtered through a 70-μm cell strainer into a 50 ml conical tube. The filter was rinsed with 5 ml of cold shaking buffer. Samples were visualized again and a portion of the suspension was centrifuged for 10 minutes at 4°C. Supernatant was gently poured off, ensuring all excess liquid was removed from the tube. Intestinal crypts were resuspended in Matrigel™ (BD Biosciences, San Jose, CA, USA) plus the growth factors R-spondin, mouse Noggin and mouse EGF (R&D Systems, Minneapolis, MN, USA) and then plated onto tissue culture plates. The Matrigel™ suspension was allowed to polymerize at 37°C for between 15 minutes and 1 hour before fresh minigut medium was supplied. Minigut medium plus growth factors was replaced every 3–4 days.

Organoids were maintained in culture for ~6–9 days before being propagated into additional wells or used for experimental procedures. Organoids were passaged by rinsing with ice cold PBS and then adding additional cold PBS and using a P1000 pipette tip to scrape the Matrigel™ from the vessel surface and break apart organoids. Intestinal organoid-Matrigel™ suspensions were pooled, spun down at 4°C for 10 minutes at 150 ***g***. Organoids were resuspended in 100–250 ml of cold PBS and passed through a syringe needle several times to further break apart crypts.

Organoids were then re-centrifuged at 150 ***g*** to pellet the suspension. Matrigel™ with growth factors was used to resuspend the crypts, which were then relayed onto tissue culture plates.

### qPCR

PER2::LUC organoids were plated into 12-well plates at the start of the experiment. Organoids for each time point were plated into a separate plate to limit manipulation or exposure to possible resetting cues. End of serum shock is indicated by circadian time 0 to compare with bioluminescent recordings performed in parallel. RNA was harvested every 4 hours over a 48-hour period using 0.25 ml/well of TRI Reagent™ RT (Molecular Research Center, Cincinnati, OH, USA). Suspensions were passed through a syringe needle several times to break up the Matrigel™ and organoids. Samples were immediately stored at −80°C until all time points were collected and could be processed. Total RNA was treated with RQ1 DNaseI (Promega, Fitchburg, WI, USA). For reverse transcription reactions, 1 μg of total RNA was used with GoScript Reverse Transcriptase (Promega) according to manufacturer’s instructions. qPCR was performed with SYBR Green Master Mix (Qiagen, Germantown, MD, USA). qPCR results were detected by using the StepOnePlus System (Life Technologies, Grand Island, NY, USA).

### Bioluminescent recordings

For Kronos Dio™ incubating luminometer experiments, PER2::LUC organoids were plated into 35-mm dishes, allowed to polymerize and either exposed to a 50% fetal bovine serum shock for 2 hours or immediately overlaid with minigut medium. Before luminometry, all samples were placed in minigut medium plus 200 μM Beetle Luciferin K^+^ Salt (Promega, Fitchburg, WI, USA). No serum shock was performed on tissue explants. For experiments performed in the absence of Matrigel™, organoids were resuspended and plated in 1.5 ml of growth medium with Beetle Luciferin K^+^ salt and 10 μM Y-27632 (ROCK inhibitor, Sigma-Aldrich, St Louis, MO, USA) to stabilize floating organoids. RU486 (Sigma-Aldrich) that had been solubilized in 100% ethanol was added directly to 50 μl of the Matrigel™-organoid suspension to a final concentration of 20 μM. For vehicle controls, an equal volume of 100% ethanol was added in the same manner. For growth factor pulse experiments, a pulse of standard growth factors (500 ng/ml R-Spondin, 100 ng/ml mouse Noggin, 50 ng/ml mouse EGF) or an equal volume of PBS (vehicle control) were added to the medium at the upslope or downslope of the second peak, with timing calculated as 20 and 32 hours after the first peak.

### R-spondin removal experiments

PER2::LUC enteroids were plated in Matrigel™ with mouse Noggin and mouse EGF. R-spondin was added to the medium instead of Matrigel™ to ensure complete washout. Samples were serum shocked for 2 hours. Serum shock was then replaced with minigut medium plus R-spondin for both the control and 24-hour washout samples, or minigut medium alone for the No R-spondin samples. After 24 hours, all samples were washed with 1 ml warm PBS then refreshed with minigut medium with Noggin and EGF. R-spondin was added back only to control samples. Brightfield images (4× magnification) were taken immediately following washout, then again on Day 3 and Day 5 to monitor viability under the three conditions using the Nikon ECLIPSE TE 200-U microscope.

### Intestinal cell lines

Nontransformed, conditionally immortalized mouse small intestinal epithelial cells (a generous gift from Robert Whitehead, Vanderbilt University, Nashville, TN, USA) and transformed Caco-2 cell lines were grown to confluence in their respective media (RPMI 1640 or DMEM/F12) at 37°C (this temperature reverts MSIE cells to a quiescent, wild-type state). At ~80% confluence, cells were shocked with 50% fetal bovine serum in medium for 2 hours, which was then replaced with standard growth medium. The start of serum shock is indicated by circadian time 0. RNA was harvested at 4-hour intervals over 48 hours using Qiagen RNeasy Mini™ kit and QIAshredder™ columns (Qiagen, Germantown, MD, USA). Briefly, RLT buffer plus 2-mercaptoethanol was used to lyse cells and a rubber cell scraper was used to release them from the tissue culture plastic according to the manufacturer’s instructions. The contents of each well were collected individually using the RNeasy protocol. Once RNA was isolated and quantified, cDNA was generated using the Superscript III First-Strand Synthesis System™ (Invitrogen, Carlsbad, CA, USA). qPCR was performed using Agilent Brilliant III Ultra-Fast SYBR QPCR Mastermix (Agilent Technologies, Santa Clara, CA, USA) with the Stratagene MX3000™ qPCR machine. The cDNA from all three samples per time point was pooled and run in triplicate on 96-well plates using *Per2* and *Gapdh* primers (IDT, Coralville, IA, USA).

### Confocal microscopy

For whole mount immunofluorescence, organoids were plated onto chamber slides and fixed in 4% paraformaldehyde for 30 minutes. Organoids were permeabilized with PBS containing Triton X-100 (0.1%) for 30 minutes and blocked using 10% goat serum for 30 minutes. Organoids were immunostained with primary antibodies specific for PER2 and LUC (SC-25363 and SC-74548, respectively; Santa Cruz) at a 1:100 dilution at 4°C overnight followed by 4°C overnight incubation with a 1:200 dilution of Alexa Fluor 633 or 488 goat anti-rabbit IgG (Invitrogen).
